# RHAMM induces progression of rheumatoid arthritis by enhancing the functions of fibroblast-like synoviocytes

**DOI:** 10.1186/s12891-018-2370-6

**Published:** 2018-12-26

**Authors:** Jing Wu, Yuan Qu, Yu-Ping Zhang, Jia-Xin Deng, Qing-Hong Yu

**Affiliations:** Department of Rheumatology and Clinical Immunology, ZhuJiang Hospital, Southern Medical University, 253 Gongye Ave GuangZhou, GuangDong, 510282 China

**Keywords:** Receptor for hyaluronan-mediated motility, Rheumatoid arthritis, Synoviocyte, siRNA

## Abstract

**Background:**

Rheumatoid arthritis (RA) is a chronic and refractory autoimmune joint disease. Fibroblast-like synoviocytes (FLS) produce inflammatory cytokines and are involved in the migration and invasion of panuus tissue, which leads to the destruction of joints in RA. Receptor for hyaluronan mediated motility (RHAMM), is known to be one of the important receptors for hyaluronic acid. It has the ability to regulate migration of fibrocytes and infiltration of inflammatory cells. Here,we explored the mechanisms of RHAMM in RAFs.

**Methods:**

Quantitative PCR and western blot were performed to test the expression of RHAMM in synoviocytes of RA patients and osteoarthritis (OA) controls. Collagen antibody-induced arthritis (CAIA) was used to investigate the RHAMM expression in mouse synovial issues. RHAMM siRNA was used to detect the function of RHAMM in FLS.

**Results:**

RA-FLS has a significantly higher expression of RHAMM than OA-FLS. Expression of RHAMM in joint synovial tissue was markedly increased in the CAIA mice compared with the controls. RHAMM silencing using SiRNA was not only decreased the production of IL-6 and IL-8, but also inhibited the migration and invasion of RA-FLS.

**Conclusions:**

RHAMM has an important role in the FLS induced modulation of inflammation and destruction of joints in RA.

## Background

Rheumatoid arthritis (RA) is an immune-mediated disorder induced by chronic and refractory autoimmunity in the synovial joints, which leads to the damage of the cartilage and bone. RA affects approximately 1% of the population globally, although its complex pathogenesis is not fully understood.[1, 2] The fibroblast-like synoviocytes (FLS) play a critical role in the progression of RA. These cells normally line the synovium, but in RA they proliferate in an uncontrolled manner and form the pannus tissue, a tumor-like structure that causes significant damage to the joints [3, 4]. In the synovial tissue, FLS secrete cytokines that induce persistent inflammation and degradation of the cartilage and bone[5–7]. Some pro-inflammatory cytokines like IL-6 and IL-8 were proposed as important factors in the pathogenesis of the synovial inflammation[8, 9]. FLS not only invades into the extracellular matrix and thus aggravates the joint damage, but it also promotes disease progression by migrating to the unaffected joints [10, 11]. Although the invasion and migration of FLS are known in RA pathology, the molecular basis of FLS activities is still undefined.

Several similarities have been observed between RA pathologenesis and tumor development, like inflammation, angiogenesis, and cell migration and proliferation. The extracellular matrix, hyaluronan (HA), and its receptor for RHAMM are implicated in the tumor progression [12], however, their role in RA is yet to be explored. RHAMM has an important role in several diseases, such as OA and ocular surface inflammation [13–15]. RHAMM interacts with all the types of cells and their functions, such as cell-cell adhesion, cell migration, cell proliferation, cell differentiation and metastasis [16]. Moreover, RHAMM aggravates the effect of CD44 deficiency on joint inflammation [17], which suggests RHAMM as an essential mediator of cell migration in RA pathogenesis. Hence, we hypothesized that RHAMM decreases the levels of IL-6 and IL-8, and is also involved in the migration of synoviocytes and in the infiltration process of inflammatory cells.

## Methods

### Human FLS culture

Synovial tissue samples were taken from the knees of three active RA and OA patients. RA patients were diagnosed according to 2010 the Rheumatoid arthritis classification criteria [18]. All OA patients were diagnosed based on American College of Rheumatology Subcommittee Guidelines for Osteoarthritis [19]. Biopsy samples were obtained during the knee joint arthroscopy. Synovial tissues were sectioned into 1–2 mm^3^ pieces and cultured with DMEM containing 10% fetal calf serum, 100 U/ml of penicillin and 100 μg/mL of streptomycin, and incubated in a humidified incubator containing 5% CO_2_. The culture medium was changed every 3–4 days. FLS was maintained as monolayers and serial passages between 3 to 6 were used for experiments described herein.

### Quantitative PCR

Total RNA was isolated from RA-FLS and OA-FLS using Trizol reagent (Invitrogen, USA) according to manufacturer’s protocols. Reverse transcription was carried out using the first-strand cDNA synthesis kit (TaKaRa, China). To assess the RHAMM mRNA, IL-6 and IL-8 expression, real-time PCR was performed using a SYBR Premix ExTaq kit (TaKaRa, China). The primers for RHAMM are listed as follows: Forward-CAGGTCACCCAAAGGAGTCTCG, Reverse-CAAGCTCATCCAGTGTTTGC, IL-6: Forward-CCGGGAACGAAAGAGAAGCT, Reverse-GCGCTTGTGGAGAAGGAGTT, IL-8: Forward-ATGACTCAGATGTGCTCTCAAAGG and Reverse-GCTTGCATCATGTCAGAGGAAATTC, β-actin: Forward-AACTACCTTCAACTCCATCA, Reverse-GCCAGACTCGTCATACTC.

### Western blot

Proteins were extracted from RA-FLS and OA-FLS with an extraction buffer. The samples were loaded on 8% polyacrylamide Tris/glycine gels and separated at 90 V for 1 h, then transferred to a nitrocellulose membrane at a setting of 90 V for 1 h. After blocking, the membrane was incubated over-night with primary Rabbit antibodies specific to RHAMM (1:10000, Santa Cruz, USA) or Mouse anti-β-actin (1:5000, Santa Cruz, USA) at 4 °C for overnight, and then incubated with Goat-anti-Rabbit HRP (1:5000, Santa Cruz, USA) or Equine-anti-Mouse HRP (1:10000, Santa Cruz, USA) respectively. After chemiluminescent staining and fixing, gel images were scanned and analyzed using image processing software (Image J).

### Collagen antibody-induced arthritis

Ten weeks-old C57BL/6 mice were obtained from the animal laboratory of Southern Medical University, Guangzhou,China. The mice were housed in a standard environment (25 °C and 12 h light/dark cycle), and the mice were fed with standard food and water ad libitum. All the animal treatments were conducted in accordance with the ethical guidelines of the National Institutes of Health Guide for the Care and Use of Laboratory Animals [20]. Experiments were approved by the Ethics Committee of Southern Medical University (license no.SCXK 2011–0015). Mice were randomly divided into two groups (*n* = 5/group), CAIA and control groups. In the CAIA group mice were injected intravenously with 150 μL (1.5 mg) of five anti-type II collagen monoclonal antibodies at day 0, and 70 μL (35 μg) LPS after 3 days. Every mouse in the control group was injected intravenously with 150 μL of 0.9% saline and 70 μL of 0.9% saline after 3 days.

### Arthritis scores

Mice were scored for arthritis from days 0 to 12 by using 5-point scale scoring system: 0 = no arthritis; 1 = mild joint deformity and swelling; 2 = moderate joint deformity and swelling; 3 = severe swelling in the toe, foot and ankle and 4 = severe inflammation.

### Histology

On day 12, all the mice were sacrificed by decapitation, and their ankle, lung and kidney tissues were collected and fixed in 4% paraformaldehyde at room temperature for 6 h, dehydrated, embedded in paraffin and sectioned. Paraffin sections were HE stained for the evaluating the morphology of the joint. After heat-mediated antigen retrieval, sections were incubated with primary antibody for RHAMM (1:50) at 4 °C for 12 h, and then with secondary antibody (1:50) at room temperature for 30 min. For antigen visualization, the sections were immersed in 3-amino-9-ethylcarbazole+substrate-chromogen for 30 min, and counter-stained with Gill’s haematoxylin for 30 min.

### The RNA interference assay

In this assay, the small interfering RNA (siRNA) for against RHAMM (AM16708, Invitrogen) and the no-target control siRNA (AM4611, Invitrogen) were used. FLS were transiently transfected with the indicated combinations of the siRNAs using Lipofectamine™ 2000 transfection reagent (Invitrogen, USA) according to the manufacturer’s recommendations. The efficiency of siRNA-mediated gene silencing was assessed using real-time quantitative PCR and Western blot.

### Enzyme linked immunosorbent assay

After treatement with RHAMM siRNA, 2 × 10^4^ RA-FLS or OA-FLS were stimulated in 24 wells using human IL-1 alpha (10 ng/ml, Invitrogen, USA) for 24 h, supernatant was collected and used for the detection of IL-6 and IL-8 levels by ELISA (Thermo Fisher Scientific, USA). The optical density (OD) of each sample was measured at 450 nm. Recombinant IL-6 and IL-8 cytokines were used as standards.

### Migration and invasion assay of FLS

#### Wound scratch assay

To demonstrate the effects of siRNA on the migratory capacity of FLS, wound scratch assay was performed. After treatment with RHAMM siRNA, 2 × 10^4^ RA-FLS and OA-FLS were seeded in 24-well plates. After serum starvation for 24 h, 200 μL pipette tip was used to make a perforation. FLS were viewed for 0–24 h. The rate of migration was calculated by measuring the distance of the wound after scratching as follows: rate of migration in % = [distance moved (migrating cell front)/total distance (wound margin)] × 100.

### Transwell migration and invasion assay

The directed chemotaxis assay was performed using transwell chambers with 8 μm pores (Corning, BD Biosciences) coated with bovine serum albumin (BSA). Briefly, 10% fetal bovine serum (FBS)/DMEM as a chemoattractant was placed in the lower chambers. A total of 2 × 10^4^ FLS were suspended in serum-free DMEM in the upper chambers for 48 h. After non-migrated cells were removed with a cotton swab, the membranes were fixed with 4% paraformaldehyde for 30 min and then stained with 0.1% crystal violet. Migration was quantified by using an optical microscope and counting the stained cells that migrated to the lower side of the filter. The number of migrated cells was presented as the migration index, and this value was calculated by normalizing relative to the media control,. For the invasion assay, similar experiments were performed using inserts coated with a Matrigel membrane matrix. Briefly,the FLS were seeded at a density of 2 × 10^4^ and grown in DMEM for 48 h. Cells that invaded through the matrix to the basolateral side of the membrane were fixed and stained. For further calculations, means values of the number of invading cells from six field-of-view images normalized relative to the control were used.

### Statistical analysis

Data are presented as the mean ± standard deviation. The Mann–Whitney U and the Kruskal-Wallis statistic tests using GraphPad Prism 5 were used to analyze the differences between groups. Differences were considered to be statistically significant when *p* < 0.05 at 95% confidence interval.

## Results

### Expression of RHAMM mRNA and protein in FLS

**Figure 1:**
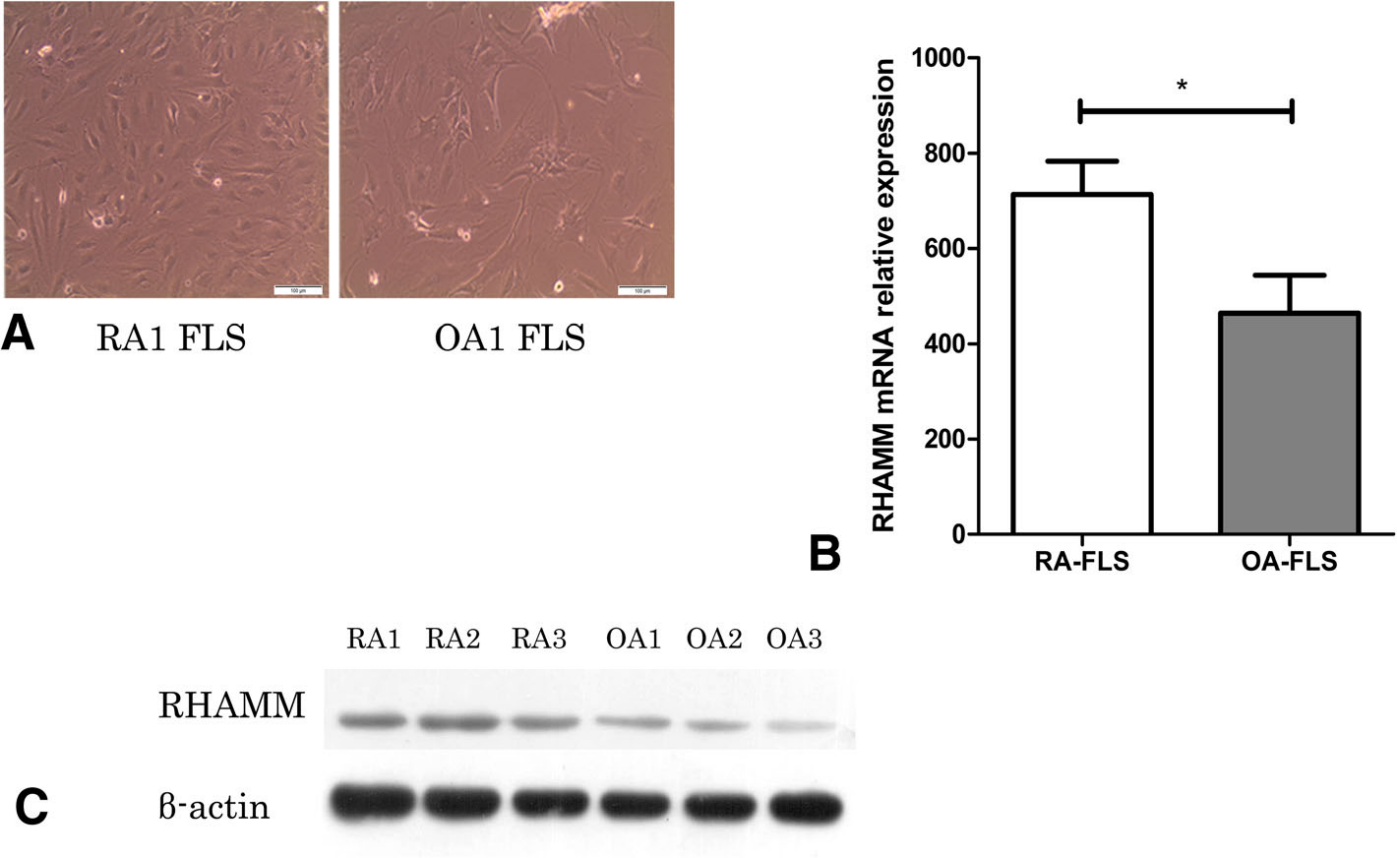
**Fig. 1** **a** RA-FLS and OA-FLS culture (*n* = 3). Each sample was analyzed twice; (**b**) FLS mRNA and (**c**) protein expressions from active RA patients were higher than OA patients. *p < 0.05, Mann–Whitney U test.

The RHAMM mRNA and protein expressions in the FLS of RA group were significantly higher (*p* < 0.05) compared with OA group (Fig. 1).

### Collagen antibody induced arthritis (CAIA) experiments

**Figure 2:**
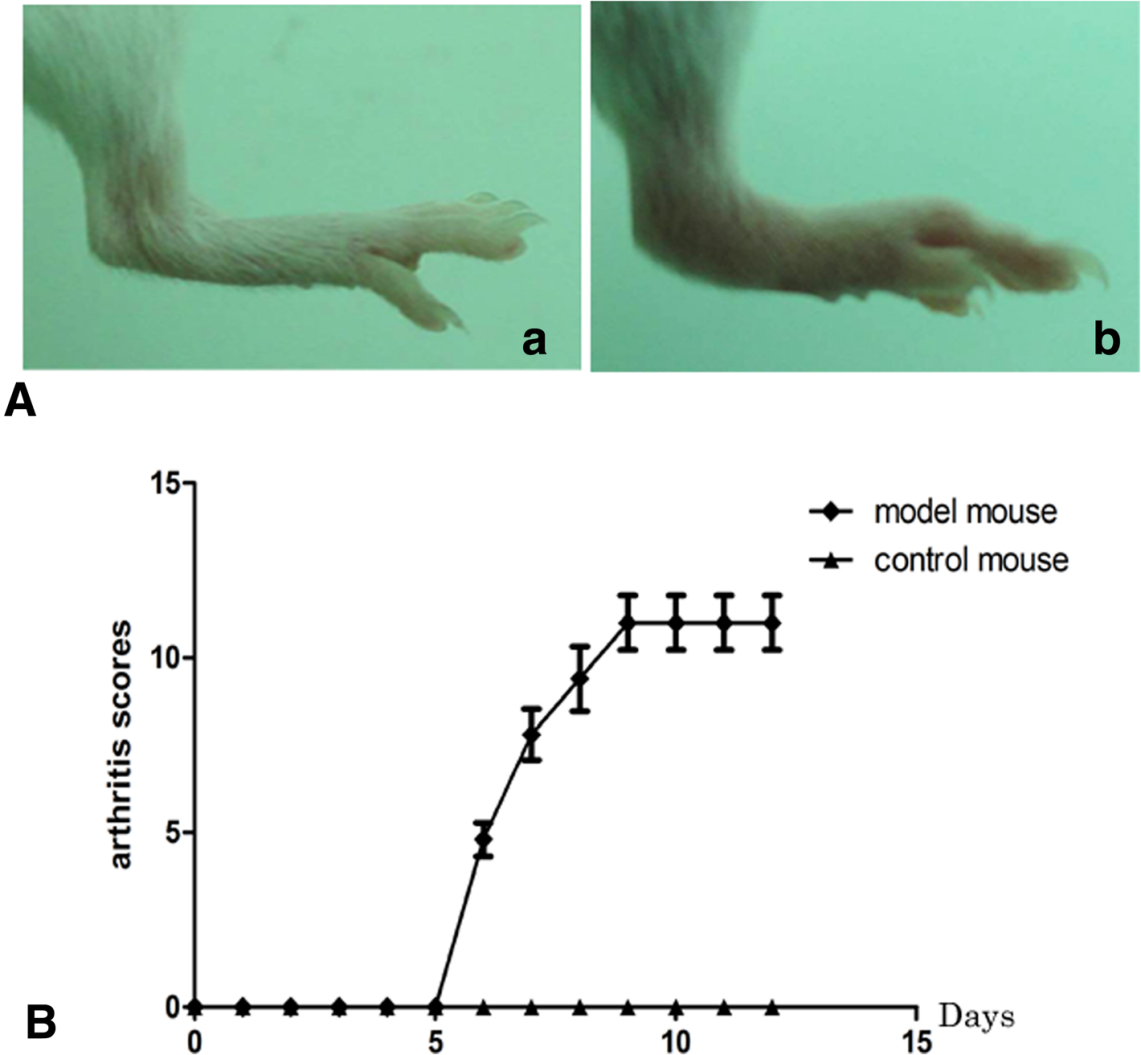
**Fig. 2** Joint swelling (**a**) and arthritis scores (**b**) in CAIA and control mice (*n* = 5/group). a: normal joint in the control mouse; b: joint swelling and inflammation of CAIA mouse on 7th day.

**Figure 3:**
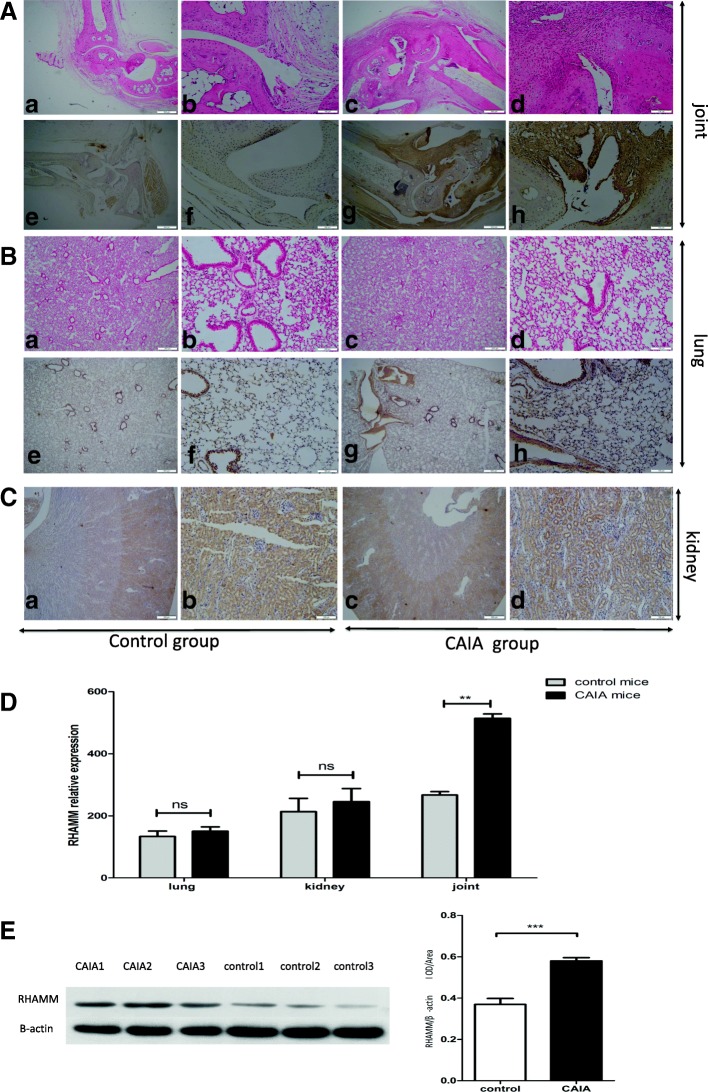
**Fig. 3** **a** HE/ Immunohistochemistry staining of joints of control mice (a, b, e, f) compared with CAIA mice (c, d, g, h). Control mice have no obvious hyperplasia of the synovial tissue and synovial tissue probably made up of 3 to 4 layers of synovial cells, low level expression of RHAMM (arrows in b and f); CAIA mice, had synovial hyperplasia and articular cartilage surface roughness, local inflammatory cell infiltration, part of synovial cells infiltrating the cartilage and bone tissue, high level expression of RHAMM (arrows in d and h). **b** No significant differences in the staining of lungs between control (a, b, e, f) and CAIA mice (c, d, g, h). **c** Immunohistochemistry staining of kidneys, control mice (a, b) compared to CAIA mice (c, d), no significant difference of RNAMM expression. **d** RT-PCR results of RHAMM expression in lung, kidney and joint sections. RHAMM was expressed in CAIA mice joints at higher levels. **e** Quantitative analyses of western-blot of joint proteins from CAIA and control mice. RHAMM was expressed higher in CAIA mice joints than control mice. ** *p* < 0.01, *** *p* < 0.001, Mann–Whitney U test.

After injection with five anti-type II collagen monoclonal antibodies and stimulated with LPS, the arthritis scores of the control and CAIA mice were 0 points on days 1 to 5 (Fig. 2). The arthritis scores of the CAIA mice increased from days 6 to 12. The ankles of the control mice didn’t show any damage on H/E staining (Fig. 3 A: a and b). On the contrary, destruction of the cartilage and bone, synovial hyperplasia, visible articular cartilage surface roughness, local infiltration of inflammatory cells, synovial hyperplasia were observed in the CAIA mice (Fig. 3 A: c and d). Immunohistochemical analysis of the ankles exhibited increased expression of RHAMM in the CAIA mice compared with the control mice, especially in the cartilage, bone and synovial membrane (Fig. 3 A: e, f and g, h).Expression of RHAMM was different in joints tissues, particularly in synovial tissues both in CAIA induced and control mice. Accordingly, elevated RHAMM expression was observed in cartilage, bone and synovial tissues compared to that in other tissues.

### Assessment of siRNA-mediated RHAMM gene silencing

**Figure 4:**
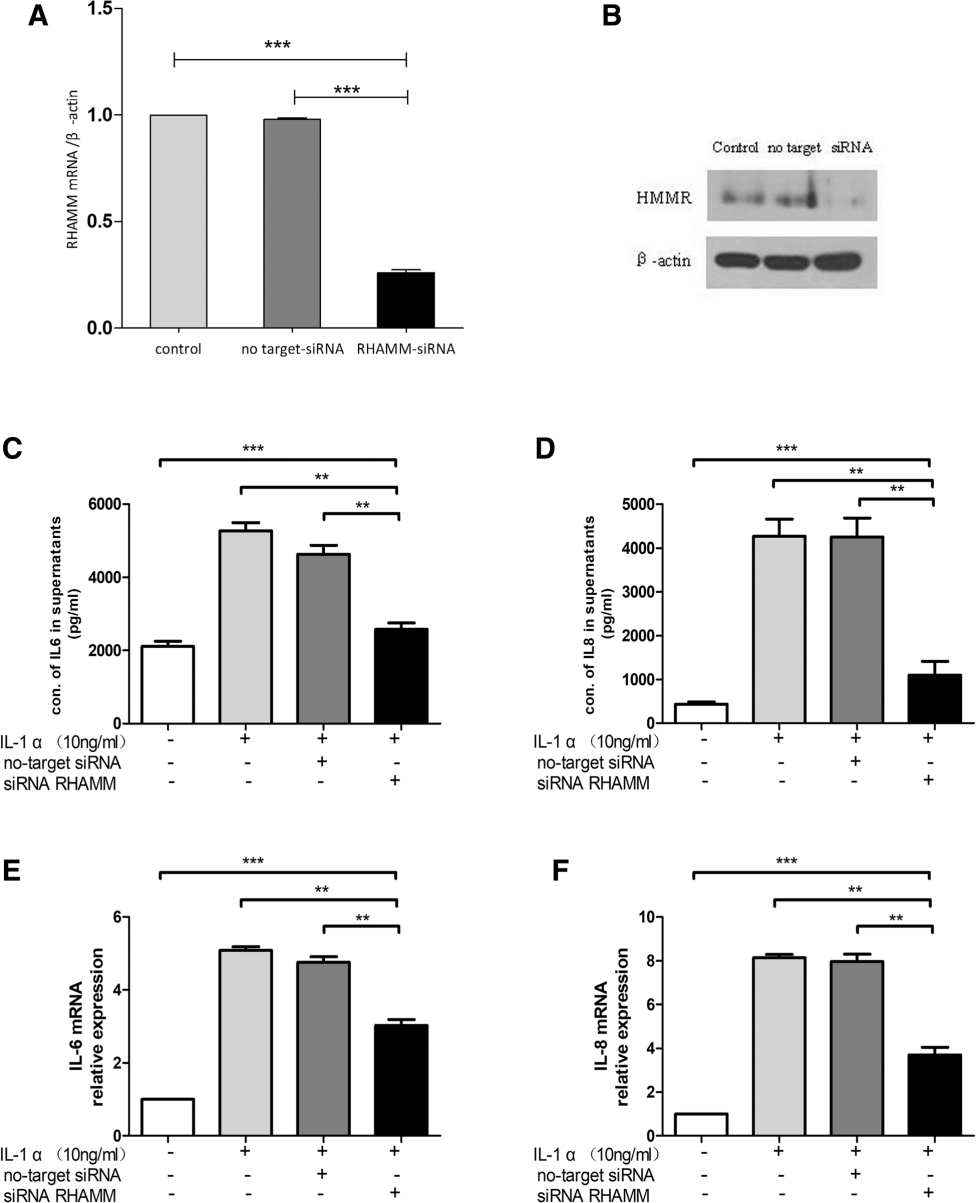
**Fig. 4** **a** Low levels of RHAMM mRNA and (**b**) protein in FLS after siRNA interference (*n* = 3). Each sample was analyzed twice); (**c**) IL-6 and (**d**) IL-8 secretions were low in siRNA treated RHAMM FLS; (**e**) Quantitative RT-PCR amplified IL-6, and (**f**) IL-8 gene expressions were lower in siRNA treated RHAMM FLS. ** *p* < 0.01, *** *p* < 0.001, Mann–Whitney U test (two groups) or Kruskal-Wallis test (more than two groups).

To evaluate the efficacy of RNA interference assay and efficacy of siRNA against RHAMM gene, RT-PCR and western blot were performed. Results showed that RHAMM expression was successfully-suppressed as a result of siRNA interference. (Fig. 4 A and B).

### Expression of IL-6 and IL-8 in synovial cells

The pro-inflammatory cytokines IL-6 and IL-8 are known key markers for FLS. We assessed the expression of IL-6 and IL-8 in the synovial cells. Both IL-6 and IL-8 levels were observed to be significantly decreased in the synovial cell culture medium (Fig. 4 C and D). In additionFurthermore, RT-PCR results showed that after interfering with the RHAMM expression, the levels of IL-6 and IL-8 were significantly decreased (Fig. 4. E and F).

### Migration and invasion

**Figure 5:**
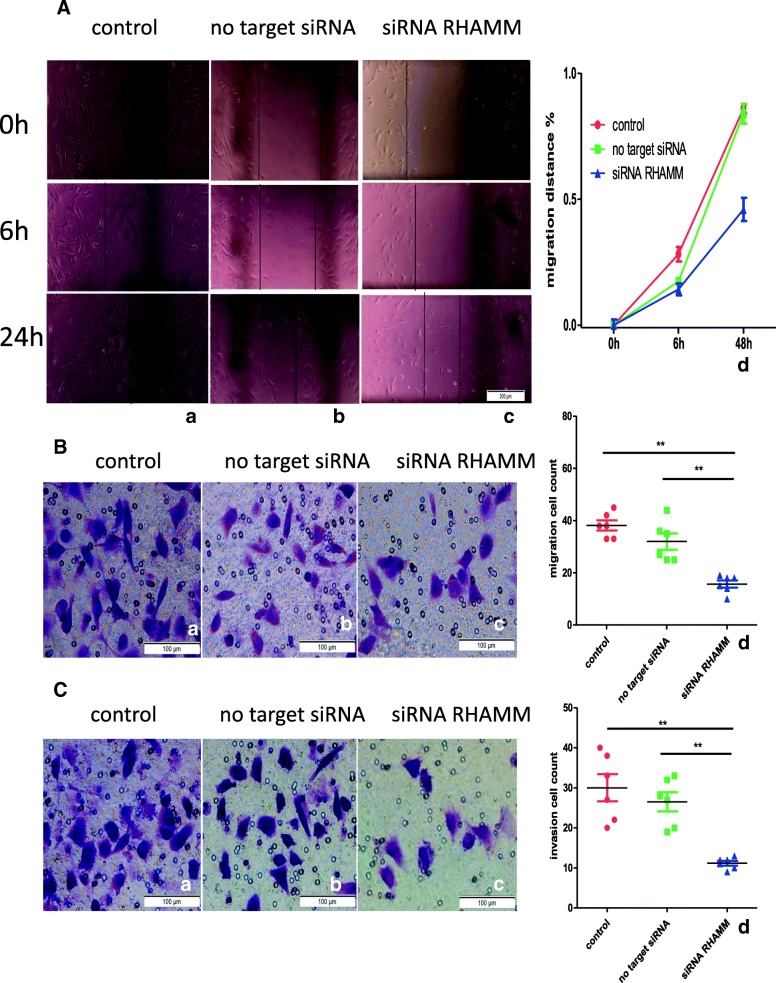
**Fig. 5** Migration and invasion results (*n* = 3). Each sample was analysed twice.(a: control group; b: no target control group; c: RHAMM siRNA group; d: statistics results). **a** Migration ability of RHAMM-siRNA group was significantly decreased compared with the control group; FLS migration cell numbers, (**b**) control and (**c**) RHAMM siRNA interference groups; ** *p* < 0.01, Mann–Whitney U test (two groups) or Kruskal-Wallis test (more than two groups).

Our results demonstrate that migration and invasion of FLS were significantly affected after the inhibition of RHAMM. In the wound scratch assay, migration ability of the RHAMM-siRNA group was decreased compared to that of the control group (Fig. 5: A). In the migration and invasion test, cell number was significantly decreased in the RHAMM-siRNA group (Fig. 5: B and C), while no obvious difference was observed in the control group and no-target interference groups.

## Discussion

The pathogenesis of RA remains to be completely elucidated, but it is well known that FLS play a key role in it. Normally,synovial fibroblasts line the joints, but in RA they are increased in numbers as part of the pannus, a tumor-like structure that causes significant joint destruction. Despite their invasive potential, not much is known about the factors and processes that mediate the invasion of FLS. HA is a ubiquitous extracellular matrix polysaccharide that belongs to the glycosaminoglycan family, which is characterized by repeating hexosamines and uronic acid units [21–23]. Low molecular weight HA is speculated to exacerbate inflammation in RA. In this context, variations in quantity and functions of HA receptors would affect the severity of inflammation in FLS, and play critical roles in maintaining the hyperplasia of synovium, pannus formation and the destruction of cartilage in RA. RHAMM is thought to be involved in the reorganization of cytoskeletal structures and movement, the essential components of the inflammatory response. Although in recent years, many studies have shown that RHAMM is involved in the migration of malignant cells and hence has an important role in immune-related diseases, so far very few data available demonstrating the role of RHAMM in RA pathogenesis. Role of RHAMM in the mechanisms of OA but not in RA was reported earlier [15]. CD44, another hyaluronan receptor, regulates cell adhesion, homing and trans endothelial migration. Most of the CD44 splice variants are more strongly expressed in the synovial membrane of RA but not OA patients [24]. Both these proteins are involved in the wound repair process and their aberrant regulation contributes to a variety of diseases including arthritis [25]. CD44 and RHAMM influence their functions reciprocally in collagen-induced arthritis. and blocking CD44 function attenuates arthritis [17]. Genetic deletion of CD44 increases arthritis severity, but blocking RHAMM function attenuates arthritis suggesting that RHAMM possibly compensates for genetic loss of CD44. Here we demonstrate higher expression of RHAMM FLS from RA patients compared with OA patients. This finding implies that RHAMM has a key role in the pathogenesis of RA.

This assumption was further validated by animal experiments. In an earlier study, visible roughness of the cartilage surface, infiltration of inflammatory cells and bone erosions were observed in the joints of CAIA mice as reported earlier [26, 27]. Immunohistochemistry results showed that RHAMM was expressed highly in the joints of CAIA mice compared to the joints of control mice. Interestingly, RHAMM is expressed in the thickened synovial membrane, cartilage and bone of the articular joints. Both RT-PCR and Western blot analyses confirm that RHAMM is indeed essential in the pathological process of RA. In RA patients, sometimes lung and kidney damages are known to be present, but in our experiments, lung and kidney damages were not observed. Our results also demonstrate organ-specificity of RHAMM in RA because of its expression in synovial tissue markedly increased compared to that in lung and kidney tissues. This specific expression pattern suggests presence of RHAMM in the synovial cells could be part of RA pathogenesis, in which RHAMM possibly regulates behavioral and functional aspects of FLS. To our knowledge, for the first time we are report that RHAMM plays an important role in the regulation of inflammation and biological behaviour of FLS isolated from RA patients. In order to investigate the functions of RHAMM, siRNA was successfully used to knock down its expression in RA-FLS resulting in decreased production of IL-6 and IL-8 cytokines, and a strong inhibitory effect on the migration and invasion of FLS.

RAFs produce IL-6 and IL-8, which are pro-inflammatory cytokines that play crucial roles in the pathophysiology of RA, and contribute to the inflammation and joint damage [28–32]. These inflammatory cytokines have been observed to degrade cartilage and bone in human patients and mouse models. Accordingly, treatment using anti-IL-6 receptor has significantly improved the clinical scores and laboratory indicators in RA patients [33, 34]. In addition, IL-6-deficient mice did not develop arthritis symptoms or joint destructions [35]. Furthermore, therapeutic targeting of cytokines in RA suggests that IL-8 may have pathogenic importance in RA [36] and IL-8 expression in RA synovial tissue associated with disease activity [37]. Decreased levels of IL-6 and IL-8 because of RHAMM expression suggests thier involvement in the modulation of FLS induced joint inflammation.

Migration and invasion of synoviocytes contribute greatly to the pathogenesis of RA [10]. Synoviocyte migration to cartilage and bone is a crucial process in aggravating of cartilage destruction in RA. FLS can invade into the extracellular matrix leading to a further aggravation of joint damage, and promote disease progression by migrating to the unaffected joints [11]. In our study, RHAMM silencing of RHAMM gene using siRNA has decreased the production of IL-6 and IL-8, and had a strong inhibitory effect on the migration and invasion of FLS. This suggests RHAMM’s ability in the modulation of joint destruction induced by RA-FLS.

## Conclusions

Our results provide important evidence for RHAMM involvement in the modulation of inflammation and joint destruction caused by RA-FLS. RHAMM controls the secretion of pro-inflammatory factors, like IL-6 and IL-8, but the exact mechanisms remain to be investigated. RHAMM is well-known for its role in the hyaluronan mediated motility pathway, which affects the functions of the immune system. Here, we observed high level expression of RHAMM in RA-FLS. RHAMM controls the invasion and migration of FLS and also modulates the secretion of IL-6 and IL-8 from FLS. Therefore, we propose involvement of RHAMM in the enhancement of inflammation and subsequent deterioration of RA.

## Abbreviations

CAIA: Collagen antibody-induced arthritis
FLS: Fibroblast-like synoviocytes
OA: Osteoarthritis
RA: Rheumatoid arthritis
RHAMM: Receptor for hyaluronan mediated motility

## References

[1] Firestein GS (2003). Evolving concepts of rheumatoid arthritis. Nature.

[2] Scott DL, Wolfe F, Huizinga TW (2010). Rheumatoid arthritis. Lancet.

[3] Bottini N, Firestein GS (2013). Duality of fibroblast-like synoviocytes in RA: passive responders and imprinted aggressors. Nat Rev Rheumatol.

[4] Filer A (2013). The fibroblast as a therapeutic target in rheumatoid arthritis. Curr Opin Pharmacol.

[5] McInnes IB, Schett G (2011). The pathogenesis of rheumatoid arthritis. N Engl J Med.

[6] Huber LC, Distler O, Tarner I, Gay RE, Gay S, Pap T (2006). Synovial fibroblasts: key players in rheumatoid arthritis. Rheumatology (Oxford).

[7] Noss EH, Brenner MB (2008). The role and therapeutic implications of fibroblast-like synoviocytes in inflammation and cartilage erosion in rheumatoid arthritis. Immunol Rev.

[8] Feldmann M, Brennan FM, Maini RN (1996). Rheumatoid arthritis. Cell.

[9] Terenzi R, Romano E, Manetti M, Peruzzi F, Nacci F, Matucci-Cerinic M (2013). Neuropeptides activate TRPV1 in rheumatoid arthritis fibroblast-like synoviocytes and foster IL-6 and IL-8 production. Ann Rheum Dis.

[10] Bartok B, Firestein GS (2010). Fibroblast-like synoviocytes: key effector cells in rheumatoid arthritis. Immunol Rev.

[11] Lefevre S, Knedla A, Tennie C, Kampmann A, Wunrau C, Dinser R (2009). Synovial fibroblasts spread rheumatoid arthritis to unaffected joints. Nat Med.

[12] Gust KM, Hofer MD, Perner SR, Kim R, Chinnaiyan AM, Varambally S (2009). RHAMM (CD168) is overexpressed at the protein level and may constitute an immunogenic antigen in advanced prostate cancer disease. Neoplasia.

[13] Naor D, Nedvetzki S, Walmsley M, Yayon A, Turley EA, Golan I (2007). CD44 involvement in autoimmune inflammations: the lesson to be learned from CD44-targeting by antibody or from knockout mice. Ann N Y Acad Sci.

[14] Garcia-Posadas L, Contreras-Ruiz L, Arranz-Valsero I, Lopez-Garcia A, Calonge M, Diebold Y (2014). CD44 and RHAMM hyaluronan receptors in human ocular surface inflammation. Graefes Arch Clin Exp Ophthalmol.

[15] Dunn S, Kolomytkin OV, Waddell DD, Marino AA (2009). Hyaluronan-binding receptors: possible involvement in osteoarthritis. Mod Rheumatol.

[16] Savani RC, Cao G, Pooler PM, Zaman A, Zhou Z, DeLisser HM (2001). Differential involvement of the hyaluronan (HA) receptors CD44 and receptor for HA-mediated motility in endothelial cell function and angiogenesis. J Biol Chem.

[17] Nedvetzki S, Gonen E, Assayag N, Reich R, Williams RO, Thurmond RL (2004). RHAMM, a receptor for hyaluronan-mediated motility, compensates for CD44 in inflamed CD44-knockout mice: a different interpretation of redundancy. Proc Natl Acad Sci U S A.

[18] Aletaha D, Neogi T, Silman AJ, Funovits J, Felson DT, Bingham CO, 3rd, et al. 2010 rheumatoid arthritis classification criteria: an American College of Rheumatology/European league against rheumatism collaborative initiative. Arthritis Rheum 2010; 62:2569–2581. 10.1002/art.27584 20872595

[19] Recommendations for the medical management of osteoarthritis of the hip and knee: 2000 update. American College of Rheumatology Subcommittee on Osteoarthritis Guidelines Arthritis Rheum. 2000;43:1905–15. 10.1002/1529-0131(200009)43:9<1905::AID-ANR1>3.0.CO;2-P 11014340

[20] Institute of Laboratory Animal Resources (U.S.). Committee on Care and Use of Laboratory Animals.: Guide for the care and use of laboratory animals. In: *NIH publication.* Bethesda, Md.: U.S. Dept. of Health and Human Services, Public Health Service: v.

[21] Ballard PL, Gonzales LW, Godinez RI, Godinez MH, Savani RC, McCurnin DC (2006). Surfactant composition and function in a primate model of infant chronic lung disease: effects of inhaled nitric oxide. Pediatr Res.

[22] Khan F, Ahmad SR (2013). Polysaccharides and their derivatives for versatile tissue engineering application. Macromol Biosci.

[23] Breitkreutz D, Koxholt I, Thiemann K, Nischt R (2013). Skin basement membrane: the foundation of epidermal integrity--BM functions and diverse roles of bridging molecules nidogen and perlecan. Biomed Res Int.

[24] Grisar J, Munk M, Steiner CW, Amoyo-Minar L, Tohidast-Akrad M, Zenz P (2012). Expression patterns of CD44 and CD44 splice variants in patients with rheumatoid arthritis. Clin Exp Rheumatol.

[25] Turley EA, Naor D (2012). RHAMM and CD44 peptides-analytic tools and potential drugs. Front Biosci (Landmark Ed).

[26] Croxford AM, Whittingham S, McNaughton D, Nandakumar KS, Holmdahl R, Rowley MJ (2013). Type II collagen-specific antibodies induce cartilage damage in mice independent of inflammation. Arthritis Rheum.

[27] Nandakumar KS, Holmdahl R (2007). Collagen antibody induced arthritis. Methods Mol Med.

[28] Miyazawa K, Mori A, Yamamoto K, Okudaira H (1998). Constitutive transcription of the human interleukin-6 gene by rheumatoid synoviocytes: spontaneous activation of NF-kappaB and CBF1. Am J Pathol.

[29] Tan PL, Farmiloe S, Yeoman S, Watson JD (1990). Expression of the interleukin 6 gene in rheumatoid synovial fibroblasts. J Rheumatol.

[30] Georganas C, Liu HT, Perlman H, Hoffmann A, Thimmapaya B, Pope RM (2000). Regulation of IL-6 and IL-8 expression in rheumatoid arthritis synovial fibroblasts: the dominant role for NF-kappa B but not C/EBP beta or c-Jun. J Immunol.

[31] Nanki T, Nagasaka K, Hayashida K, Saita Y, Miyasaka N (2001). Chemokines regulate IL-6 and IL-8 production by fibroblast-like synoviocytes from patients with rheumatoid arthritis. J Immunol.

[32] Nanki T, Nagasaka K, Hayashida K, Saita Y, Miyasaka N (2001). Chemokines regulate IL-6 and IL-8 production by fibroblast-like synoviocytes from patients with rheumatoid arthritis. J Immunol.

[33] Genovese MC, McKay JD, Nasonov EL, Mysler EF, da Silva NA, Alecock E (2008). Interleukin-6 receptor inhibition with tocilizumab reduces disease activity in rheumatoid arthritis with inadequate response to disease-modifying antirheumatic drugs: the tocilizumab in combination with traditional disease-modifying antirheumatic drug therapy study. Arthritis Rheum.

[34] Nishimoto N, Yoshizaki K, Miyasaka N, Yamamoto K, Kawai S, Takeuchi T (2004). Treatment of rheumatoid arthritis with humanized anti-interleukin-6 receptor antibody: a multicenter, double-blind, placebo-controlled trial. Arthritis Rheum.

[35] Yokota K, Miyazaki T, Hirano M, Akiyama Y, Mimura T (2006). Simvastatin inhibits production of interleukin 6 (IL-6) and IL-8 and cell proliferation induced by tumor necrosis factor-alpha in fibroblast-like synoviocytes from patients with rheumatoid arthritis. J Rheumatol.

[36] Koch AE, Kunkel SL, Burrows JC, Evanoff HL, Haines GK, Pope RM (1991). Synovial tissue macrophage as a source of the chemotactic cytokine IL-8. J Immunol.

[37] Kraan MC, Patel DD, Haringman JJ, Smith MD, Weedon H, Ahern MJ (2001). The development of clinical signs of rheumatoid synovial inflammation is associated with increased synthesis of the chemokine CXCL8 (interleukin-8). Arthritis Res.

